# Emotional responses to the experience of cancer ‘alarm’ symptoms

**DOI:** 10.1002/pon.3964

**Published:** 2015-09-11

**Authors:** K. L. Whitaker, S. Cromme, K. Winstanley, C. Renzi, J. Wardle

**Affiliations:** ^1^ School of Health Sciences, Faculty of Health and Medical SciencesUniversity of SurreyUK; ^2^ Health Behaviour Research Centre, Epidemiology and Public HealthUniversity College LondonUK

## Abstract

**Objective:**

To qualitatively explore associations between emotional responses to experience of cancer ‘alarm’ symptoms and help‐seeking in a community sample of adults.

**Method:**

Interviewees (n = 62) were recruited from a community sample (n = 2042) of adults aged ≥50 years, who had completed a health survey that included a list of cancer alarm symptoms. Participants who had reported an alarm symptom both at baseline and 3‐month follow‐up (n = 271), and who had consented to contact (n = 215), constituted the pool for invitations to interview.

**Results:**

Over a third of participants (37%) described an emotional response to their symptom experience. In all these cases, there was evidence of awareness of the risk of cancer. Emotional responses were usually either classified as mild (‘worry’) or severe (‘fear’). Worry was often described in the context of a desire to seek medical help, either to rule out cancer or to minimise patient delay. In contrast, the ‘fear’ group described associations with death, the perceived incurability of cancer, and the consequence of a cancer diagnosis. Where the emotional reaction was fear, medical contact was seen as something to be avoided either because it had no value or because it was preferable not to be told a diagnosis.

**Conclusion:**

In this community sample, worry about the possibility of cancer was associated with help‐seeking, either for reassurance or as part of a ‘sensible’ strategy to deal with the risk. In contrast, fear was associated with avoiding help‐seeking or even thinking about cancer, which could lead to prolonged help‐seeking intervals. © 2015 The Authors. *Psycho‐Oncology* published by John Wiley & Sons Ltd.

## Introduction

Emotional responses to symptoms are likely to be an important influence on behavioural responses 1. The symptom appraisal process (‘time from detection of a bodily change to perceiving a reason to discuss symptoms with a health care practitioner’) 2 is considered a key step in relation to achieving earlier presentation, earlier cancer diagnoses, and thereby better cancer outcomes 3. It is possible that a better understanding of the role of emotional responses could help explain some of the variation in help‐seeking.

There have been many studies in which patients/people diagnosed with cancer are asked to reflect back on how they responded to their first symptom, often focusing on delay in help‐seeking. In a qualitative synthesis, fear was identified as a key reason for delay, second only to not recognising the significance of the symptom 4. Predominantly the fear was of cancer itself or its treatment or side effects, but also included aspects of the help‐seeking process (e.g. being seen as neurotic). Other studies have also identified fear of investigations, and fear of diagnosis or treatment as risk factors for patient delay in upper gastrointestinal, colorectal, urological, gynaecological, and lung cancers 5. However, fear is not always found to be a deterrent. In one study, patients who recalled being more anxious when they detected a breast symptom tended to have sought help more promptly than those who had less of an emotional response 6. Another study, focusing specifically on fear intensity concluded that higher levels of fear facilitated rather than hindered help‐seeking 7. Finally, a recent systematic review of studies with patients also highlighted the mixed impact of emotions on time to presentation, concluding that more emotion‐focused research was needed in order to unravel its complexity 8.

A different approach to investigating the role of fear is to examine emotional reactions to symptoms in general population samples, and link these to help‐seeking. This avoids the retrospective bias inevitable in clinical recall studies, and also includes people who have symptoms but do not ultimately receive a cancer diagnosis. In a population‐based survey asking about help‐seeking for hypothetical symptoms (‘If you had a symptom you thought might be serious, what might put you off help‐seeking’), worry about what the doctor might find was the most commonly endorsed emotional barrier, and perceiving more barriers to help‐seeking was associated with longer anticipated delay 9. However, use of reactions to ‘hypothetical’ symptoms and ‘anticipated’ help‐seeking has its own problems in that people may not be good at predicting how they would really act if the situation arose.

One alternative is to study people's emotional responses to symptoms they are actually experiencing and link them to help‐seeking. Recent research with a community sample suggested that people who were afraid of the consequences of seeking help (e.g. investigations or treatment) tended to avoid contacting their doctor for a recently experienced cancer ‘alarm’ symptom, but paradoxically, awareness of a possible link with cancer (which is usually linked with some degree of fear) tended to promote help‐seeking 10. However emotional responses to symptoms were not explored in any depth.

The aim of the present study was therefore to explore emotional reactions in a qualitative study of a sample of adults reporting persistent cancer alarm symptoms, and examine associations with past or planned help‐seeking.

## Methods

### Participant selection and recruitment

**Figure 1:**
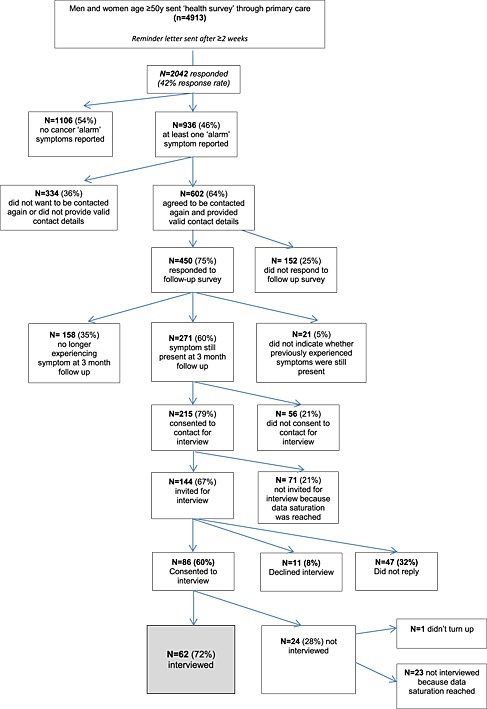
Flowchart of recruitment

Interviewees were recruited from participants (n = 2042) from a large mailed ‘health survey’ conducted through four General Practices in England across London, the South East, and the North West of England, which had been mailed to 4913 individuals aged ≥ 50 years in October 2013. Participants reporting at least one cancer ‘alarm’ symptom (n = 936) were sent a follow‐up survey after 3 months to capture persistent symptoms. Those who were still experiencing the symptom at the 3‐month follow‐up (n = 271), and who consented to contact (n = 215), constituted the pool from which we invited people to interview. We invited the first 144 participants meeting our criteria, and obtained a response rate of 60% (86/144). Interviews ceased once data saturation was achieved (n = 62) (Figure 1). Interviews were conducted as soon as possible after participants returned the follow‐up health survey, and all were conducted within 4 weeks of receipt of the survey. The study materials and protocol were approved by NHS London Bridge Research Ethics Committee (Reference: 11/LO/1970).

### Symptoms from the ‘health’ survey

Ten cancer ‘alarm’ symptoms were taken from the Cancer Awareness Measure (CAM) 11 and represented symptoms relevant to a range of cancers. We added abdominal bloating and breast changes from the ovarian and breast cancer specific versions of the CAM 12, 13 and blood in urine and rectal bleeding which were at the time being highlighted in the media *Be Clear on Cancer* campaigns designed to raise awareness of bladder and bowel cancer 14.

### Interview

Interviews were carried out during a period where people's symptom experiences were ongoing, and were intended to capture current appraisal, attribution, and decision‐making. Interviews were conducted at participants' homes (n = 8), at UCL offices (n = 15), or over the telephone (n = 39), as the participant preferred, by CR, KW, and KeW. Participants were asked to talk about various aspects of their symptom(s) including when they first noticed it, their thoughts and feelings about it over time, and any action(s) they had taken. The word ‘cancer’ was not used by the interviewer unless the participant mentioned it, to avoid biasing sense‐making and attribution accounts. After the initial narrative account, a semi‐structured topic guide was used to ensure that emotional responses were explored in sufficient detail. We used prompts to ask about emotional responses if they did not arise spontaneously, such as mentioning the experience of other interviewees (e.g. ‘*some people have mentioned that they avoid seeing the GP because they are worried about what the GP might tell them, did this ever apply to you*’). Interviews lasted on average 42 min (range 22–66 min).

### Analysis

Interviews were digitally recorded and transcribed verbatim by freelance transcribers. KW and KeW checked all transcriptions against portions of each recording and found them to be accurate. SC and KW read and re‐read the transcripts, and agreed a coding frame. Transcripts were coded, managed, and analysed using NVivo 9.0 software (QSR International Pty Ltd. 2010). Framework analysis 15 was used to create a matrix to organise the data. Data were summarised in sub‐themes under the broader themes of ‘Worry’ and ‘Fear’ (Box 1). Emerging sub‐themes were discussed in frequent meetings, and agreed by all the co‐authors. An iterative process of revisiting the original transcripts was used to ensure that the final interpretation was representative of participants' accounts. Demographic and basic frequency information was analysed using SPSS Version 21.0.

## Results

The average age of participants was 64.5 years (SD = 9.3). Both sexes were represented (47% women, 53% men). Most (92%) were White British (n = 57), and 45% (n = 28) had university‐level education. This was comparable to the demographics of the wider survey sample (n = 2042). The most common symptom reported in the interview sample was persistent cough/hoarseness (34%), and there were no reports of breast changes (Table 1). People were most likely to have sought help for persistent unexplained pain (73%).

**Table 1:** Type of symptoms and help‐seeking reported in the past 3 months in the interview sample

Symptom	Interviewees reporting symptom n (%)*	Interviewees who had contacted the GP about the symptom n (%)**
Persistent cough/hoarseness	17 (27.4)	6 (35.3)
Change in bladder habits	15 (24.2)	7 (46.7)
Change in bowel habits	12 (19.4)	4 (33.3)
Abdominal bloating	16 (25.8)	6 (37.5)
Unexplained pain	12 (19.4)	7 (72.7)
Change in mole	7 (11)	2 (28.6)
Rectal bleeding	4 (6.5)	2 (50)
Unexplained lump	4 (6.5)	1 (25)
Difficulty swallowing	6 (9.7)	1 (20)
A sore that does not heal	5 (8.1)	1 (20)
Unexplained weight loss	3 (4.8)	1 (50)
Blood in urine	2 (3.2)	1 (50)
Unexplained bleeding	2 (3.2)	0 (0)

* Denominator is number of people in the interview sample (n = 62).

** Percentages may not sum because of missing data.

Of sixty‐two people interviewed, 23 (37%) described at least one emotional response to their symptom. In all these cases, participants were aware of a possible link with cancer. Emotional responses were invariably in the domain of worry/anxiety/fear and tended to be clearly identifiable as mild and more cognitive (which we termed ‘worry’) or severe and highly affectively charged (which we termed ‘fear’).

**Table:** Box 1 Emotional responses to cancer ‘alarm’ symptoms

Worry	Fear
• Possibility of cancer	• Association with death
• Symptom seriousness	• Incurability of cancer
• Consequences of late diagnosis	• Negative views of treatment
	• Preference for remaining ignorant of a diagnosis (‘Ignorance is bliss’)

### Worry

#### Worry about cancer/symptom seriousness

The most commonly reported emotional response in relation to cancer ‘alarm’ symptoms was mild worry, with people questioning whether their symptom might be serious or indicative of cancer; although mostly they did not expect a cancer diagnosis. Help‐seeking was often described as seeking reassurance ‘*that it isn't something serious*’ (P42, F, persistent change in bowel habits):

‘*I just wanted reassurance that it wasn't a cancerous thing. When she said that it wasn't, then that was okay*’ (P43, F, change in the appearance of a mole).

‘*Well, I think most intelligent people going to the doctor have some sort of idea of what they think is wrong with them when they go, and often they are hoping for confirmation and to rule out that it's not something else more worrying*’ (P21, F, persistent change in bowel habits, abdominal bloating, change in the appearance of a mole).

#### Worry about later diagnosis

Another issue was the implication of delaying help‐seeking if the symptoms were because of cancer. Again participants in this group reported only relatively low key emotional responses: ‘*if you catch things early, quite often you can get cured, whereas if you delay and delay and delay, you've past a point of no return*’ (P10, M, persistent cough). This type of response to ‘alarm’ symptoms appeared to be a rational and considered approach to getting it ‘*caught as early as I can*’ (P11, M, persistent change in bowel habits).

### Fear

A small number of participants described a much more affectively charged emotional response to their symptoms, which we termed ‘fear’. This included fear of death, the incurability of cancer, or fear about the consequences of a cancer diagnosis such as treatment or dependence on others. People reporting fear often appeared uncomfortable both talking about cancer and describing their feelings.

#### Association with death

Participants highlighted the urge to delay the awareness of mortality and death. When one participant was asked why he was scared about his symptom potentially indicating cancer, he explained:

‘*Because my feelings of immortality might disappear quite suddenly. I mean, it's the big “C”, that's what worries people. Because it's cancer and cancer kills*’ (P22, M, persistent cough).

‘*I was trying not to think of cancer, although it's, I suppose, you know, as human beings we often think one thing. But I was trying not to, I was trying to be positive or to… as I said, I had also this experience of my friend who just died of that, so it was a bit, if you like, in the back of my mind, but I was trying not to let it influence me too much*’ (P19, F, unexplained weight loss, persistent cough).

#### Fear of the incurable

People also described feeling sceptical about what could be done if they were diagnosed with cancer because there's ‘*no guarantee of a cure*’ (P16, F, persistent change in bladder and bowel habits, abdominal bloating).

‘*And I suppose the other thing about cancer, any cancer, is cure rate is improving, but it's still… I'm not sure. I mean, different cancers have different treatment success rates, but, you know, you've still got a, sort of, one in two, one in three, one in four chance, depending on what the cancer is, of not being treated successfully*’ (P22, M, persistent cough).

‘*I'm kind of expecting that there won't be much that anybody can do about it*’ (P56, M, persistent change bladder habits).

He went on to illustrate all aspects of cancer fear in his response: ‘*I mean, the cancer with a capital C, the bête‐noir element, the fear of the unknown and the incurable, that's where the fear comes from*’ (P56, M, persistent change in bladder habits).

#### Fear of treatment

Some participants specifically described fear of the treatment they would receive if they did receive a cancer diagnosis.

‘*If it was something like cancer, something major, and then the treatment, obviously, can be awful*’ (P40, F, persistent change in bladder habits, persistent difficulty swallowing, persistent cough, rectal bleeding).

This also extended to how the treatment would impact on their lifestyle, with many expressing concern over imposing themselves on others, and becoming a burden.

‘*I have to be honest, that is a fear in me. I don't want to be dependent. It's my independence thing. I think if I got old and, sort of, incapacitated, couldn't look after myself, I don't think I'd like that at all. I don't think so. I don't know what I'd do, to be honest. With that, I do put my head in the sand and try not to think about it*’ (P57, M, persistent cough).

#### Ignorance is bliss

Many participants described not wanting to seek medical advice because ‘*ignorance is bliss*’ (P33, F, change in the appearance of a mole, persistent change in bowel and bladder habits, persistent unexplained pain). In general, this response was related to concern about getting bad news: ‘*being frightened of what it may be*’ (P54, F, persistent unexplained pain). People often used alternative terminology to describe the possibility of cancer (the big ‘C’, ‘it’, and ‘the big demon’), ‘*because it's a horrible word*’ (P35, F, 55, persistent change in bowel habits, abdominal bloating).

‘*I'm not worried about the investigation. I might be worried about the outcome of the investigation*’ (P41, M, persistent change in bladder habits, persistent cough).

‘*I'd rather not know. Well, you might have something serious. I'd rather not know*.’ (P10, M, persistent cough).

‘*I suppose you are just frightened of what it might be. That's all, really, basically. I don't know, it's just the way I feel, I suppose. It is a bit frightening when you are not sure what it might be and you think it could something not very nice. I suppose that's just the way that I am, the way I think, I suppose*’ (P54, F, unexplained persistent pain).

One participant rationalised this further and weighed up the idea that living with the possibility of a cancer diagnosis was not as bad as knowing for certain that one has cancer:

‘*I've rationalised it to myself by saying, “Well, if I'm going to live with it then I'd rather not be living in fear of when it's going to strike me down. I'd rather just be living with the vague suspicion that perhaps it might be but it's more likely to be an enlarged prostate.” So I'm hiding fright, to a certain extent*’ (P56, M, persistent change in bladder habits).

## Discussion

In this community sample of people experiencing ongoing cancer ‘alarm’ symptoms, we observed two distinct groups of emotional response. One we called ‘worry’, and this was associated with concern about the possibility of cancer, and the potential for late diagnosis. In this group, the emotional response seemed to be mild, and in general these participants appeared to feel that they were capable of coping with a cancer diagnosis, although partly this could be because they typically perceived it as a relatively low probability. Those in the ‘fear’ category were clearly frightened about the implications of receiving a cancer diagnosis, associating it with incurability and death, or fearing treatment and the impact of a cancer diagnosis on daily life. By and large, they tended to avoid thinking about cancer and avoided contact with doctors that could result in such a diagnosis.

Lower‐level emotional responses such as worry have previously been reported by oral cancer patients, who rarely recalled distress in relation to their initial symptoms, but when they did describe it as general worry or concern 16. The fearful responses matched previous findings with patients/people diagnosed with cancer where fear was described as a deterrent to help‐seeking because of feeling unable to cope with a cancer diagnosis, or dreading treatment and the impact on daily life 4, 5.

Unlike some suggestions from previous work 7, we found little evidence that high levels of cancer fear triggered help‐seeking. Rather, high levels of emotional response were associated with avoidance, both in terms of the cancer discourse in general, and in terms of help‐seeking. It was the lower levels of emotional response (worry or concern) that were mentioned as promoting help‐seeking, either to provide reassurance or to do the ‘sensible thing’ in case they did have cancer.

One possibility, as has been found in studies of fear in cancer screening uptake, is that the quality of the emotional response is more important than quantity of emotion 17. In Vrinten et al.'s (2015) study, quality of fear was assessed in terms of which specific fear item was endorsed. One item specifically mentioned frequent worry, and that was associated with a higher chance of screening attendance. Another item mentioned feeling uncomfortable thinking about cancer, and that was associated with lower attendance; the worry item was interpreted as more ‘cognitive’ and the ‘uncomfortable’ item as more visceral. The present study results fit somewhat with this model and may also help explain previous mixed findings in relation to the role of emotions in time to presentation 8. Worry – even frequent worry – motivated help‐seeking partly as a means of alleviating it. Fear, in contrast, appeared to be associated with participants feeling uncomfortable thinking or talking about cancer, which mostly they regarded as an almost untreatable condition.

The Extended Parallel Process Model 18 is another potential framework. Participants who felt competent to deal with the threat (i.e. by contacting a doctor) were using a ‘danger control’ approach to manage or reduce the risk. In this group, it would be predicted that even if worry were higher, it would probably motivate even more rapid help‐seeking. In contrast, those who felt that the threat could not be controlled (e.g. cancer was seen as incurable) adopted ‘fear control’ behaviours, preferring not to discuss the issue directly or to confront further evidence of the disease by interacting with doctors. In this group higher fear would be predicted only to make help‐seeking less likely.

Some aspects of the emotional response to symptoms, which we termed fear (e.g. fear of the incurability of cancer), overlap with the concept of cancer ‘fatalism’. Broadly speaking, fatalism denotes the expectation that death cannot be avoided or postponed when cancer is diagnosed 19. Fatalistic beliefs such as ‘people die when it is their time to die and nothing can change that’ were recently found to be associated with higher risk of advanced stage at diagnosis of lung and colorectal cancer; strongly implicating delay in presentation 20. This finding emphasises the importance of understanding the subtleties of emotional reactions to the possibility of a cancer diagnosis to achieve the goal of diagnosing cancer earlier.

This is the first community study to involve an in‐depth exploration of emotional responses to symptoms suggestive of cancer. Although a minority of people reported an emotional response in relation to their symptoms, this response could have a significant impact on help‐seeking. A particular strength is that we interviewed people who were still experiencing their ‘alarm’ symptoms, which meant we avoided discussing fleeting or self‐correcting symptoms and that the symptom experience was a ‘real‐time’ event. A limitation of the study was that, inevitably, we only captured emotional responses of those willing to share their symptom experiences. The response rate for the original survey was higher than found in some previous symptom research (42%) 21, 22, and of that group, 60% agreed to the interview study; nonetheless, a significant proportion of the target population were not represented. Because some people find it difficult to describe fearful responses, and fear is also often related to avoidant responses (e.g. avoiding cancer‐related information) 23, those who were most frightened may have been the least likely to take part in the research. However, the recruitment method may have offset this to some extent, because both the survey and interview were conducted without mentioning the word ‘cancer’. It has also recently been reported that women and people from lower SES or non‐white ethnic backgrounds are disproportionally affected by cancer fear 24, so we may have missed out on that group disproportionately. Future work could explore how emotional responses vary by socio‐demographic groups. Another avenue for future work is to explore how emotional responses might vary according to symptom type. One possibility would be to focus on specific cancers where it is well‐established that late presentation is an issue (e.g. breast cancer) 25. It might also be useful in future studies to ask people what could be done to help reduce their fear.

In conclusion, in this community sample of men and women over 50 years old, we found two distinct levels of emotional response to experiencing ongoing cancer ‘alarm’ symptoms, with some individuals describing what we called ‘worry’, which was typically low level at the point of interview, while others described ‘fear’ although were often reluctant to elaborate. Those who were worried described what seemed like ‘sensible’ actions to control risk, seeking medical advice either with the expectation of reassurance or to avoid a late diagnosis. Those we described as experiencing fear alluded to the likely failure and unpleasant features of treatment, and ultimately death. Strikingly, this group attempted to avoid the cancer discourse altogether and often required direct questions. These findings support ongoing public health initiatives that open the cancer discourse and promote cancer awareness. It may also be useful to incorporate positive messages about advances in treatment toxicity and outcomes to address the negative consequences of fearful responses.

## Conflict of interest

None declared.
